# The Association of Periodontal Treatment and Decreased Pneumonia: A Nationwide Population-Based Cohort Study

**DOI:** 10.3390/ijerph17010356

**Published:** 2020-01-05

**Authors:** Li-Chiu Yang, Yih-Jane Suen, Yu-Hsun Wang, Tai-Chen Lin, Hui-Chieh Yu, Yu-Chao Chang

**Affiliations:** 1 School of Dentistry, Chung Shan Medical University, Taichung 40201, Taiwan; licioyang@hotmail.com (L.-C.Y.); suenyihjane1@gmail.com (Y.-J.S.); linperi@tmd.ac.jp (T.-C.L.); yujessica7@gmail.com (H.-C.Y.); 2 Department of Dentistry, Chung Shan Medical University Hospital, Taichung 40201, Taiwan; 3 Department of Medical Research, Chung Shan Medical University Hospital, Taichung 40201, Taiwan; cshe731@csh.org.tw

**Keywords:** pneumonia, periodontal treatment, chronic periodontitis, nationwide population, cohort study, Taiwan

## Abstract

Pneumonia is a common respiratory infectious disease that involves the inflammation of the pulmonary parenchyma. Periodontal disease is widespread and correlated with pneumonia. However, the relationship between periodontal treatment and clinical infectious outcomes in patients with pneumonia has remained undetermined. The aim of this study was to investigate the association between periodontal treatment and the risk of pneumonia events in the Taiwanese population. A nationwide population-based cohort study was conducted using data from the Taiwanese National Health Insurance Research Database (NHIRD). A total of 49,400 chronic periodontitis patients who received periodontal treatment from 2001 to 2012 were selected. In addition, 49,400 healthy individuals without periodontal diseases were picked randomly from the general population after propensity score matching according to age, gender, monthly income, urbanization, and comorbidities. The Cox proportional hazard regression analysis was adopted to assess the hazard ratio (HR) of pneumonia between the periodontal treatment cohort and the comparison cohort. The average ages of the periodontal treatment and comparison groups were 44.25 ± 14.82 years and 44.15 ± 14.5 years, respectively. The follow up durations were 7.66 and 7.41 years for the periodontal treatment and comparison groups, respectively. We found 2504 and 1922 patients with newly diagnosed pneumonia in the comparison cohort and the periodontal treatment cohort, respectively. The Kaplan–Meier plot revealed that the cumulative incidence of pneumonia was significantly lower over the 12 year follow-up period in the periodontal treatment group (using the log-rank test, *p* < 0.001). In conclusion, this nationwide population-based study indicated that the patients with periodontal treatment exhibited a significantly lower risk of pneumonia than the general population.

## 1. Introduction

The oral environment is a very complex microenvironment consisting of multiple bacteria and their associated biofilms which can start a series of immune inflammatory reactions leading to the destruction of the periodontium [1]. Multiple infections resulting from poor oral health could also evoke a systemic response [2]. Many studies have provided scientific evidence suggesting that periodontitis could affect an individual systemically, contributing to cardiovascular disease [3], diabetes [4], and respiratory disease [5].

Pneumonia is a common respiratory infectious disease of the pulmonary parenchyma caused by bacteria, mycoplasma, viruses, fungi, or parasites. Bacterial pneumonia is a common and significant cause of mortality and morbidity in human populations [6]. Pneumonia has been the third most common cause of death in Taiwan in 2018 [7]. Dental biofilms and periodontal inflammation appear to be risk factors for the development and/or progression of pneumonia [8]. In addition, studies have reported that respiratory pathogens preferentially colonize the teeth or dentures rather than soft tissue in the oral cavity [9,10]. This indicates that dental biofilm acts as a reservoir for respiratory pathogens. Therefore, oral interventions aimed at reducing the bacteria count may result in a reduced incidence of pneumonia.

However, there is limited information on whether periodontal treatment could reduce pneumonia risk. We hypothesized that chronic periodontitis patients with periodontal treatment have a lower risk of developing pneumonia than the general population. In this study, we conducted a nationwide population-based study using the Taiwanese National Health Research Insurance database (NHIRD) to investigate the impact of periodontal treatment on pneumonia risk, with a cohort study design.

## 2. Materials and Methods

### 2.1. Data Source

The compulsory National Health Insurance program, which has operated since 1995, enrolled nearly the whole Taiwanese population in 2014 [11]. The Longitudinal Health Insurance Database 2010 (LHID2010), a part of the NHIRD, was used in this cohort study. LHID2010 comprises 1,000,000 randomly sampled beneficiaries enrolled from the National Health Insurance (NHI) program in 2010. Collected data such as registration information and dental and medical records on these individuals were described previously [12,13,14]. The NHIRD identifies diseases on the basis of the International Classification of Diseases, Ninth Revision, Clinical Modification (ICD-9-CM). The accuracy and validity of NHIRD diagnosis codes have been documented. This study was approved by the Chung Shan Medical University Hospital institutional review board (CS2-15071). This study also complies with the guidelines of strengthening the reporting of observational studies in epidemiology (STROBE).

### 2.2. Study Sample

The data for this cohort study were retrieved from LHID2010. All patients aged ≥20 years with chronic periodontitis diagnosed by ICD-9-CM code 523.4 were identified. The periodontal treatment group comprised individuals who had received periodontal treatment within 1 year following their diagnosis of chronic periodontitis. To validate the periodontal treatment sourced from LHID2010, we identified periodontal treatment cases by NHI system treatment codes (scaling 91003, 91004; subgingival curettage/root planning 91006, 91007, 91008; periodontal flap surgery 91009, 91010). Only patients with at least three outpatient service claims were included. Patients diagnosed with chronic periodontitis before 2001 were also excluded. The newly diagnosed chronic periodontitis patients with periodontal treatment from 2001 to 2012 were used as the periodontal treatment cohort. The index date was defined as the first time of treatment in the 1 year following the patient’s diagnosis of chronic periodontitis. A comparison cohort was randomly selected from those participants who were never diagnosed with any type of gingival and periodontal diseases between 2000 and 2013 (ICD-9-CM: 523) in order to ensure the accuracy of the periodontal health information. 

### 2.3. Covariates and Matching

The baseline variables included age, gender, monthly income, urbanization, and comorbidities. The monthly income data used a NT $20,000 interval to distinguish income levels according to the basic wage (NT $19,047) announced by the Ministry of Labor in 2013 [15]. Therefore, monthly income was categorized as follows: <NT $20,000, NT $20,000 to NT $40,000, and >NT $40,000. The degree of urbanization was clustered into urban, suburban, and rural based on population density, the population ratio of people with college-or-above educational levels, the population ratio of elderly people over 65 years old, the population ratio of agriculture workers, and the number of physicians per 100,000 people [16]. The comorbidities included hypertension (ICD-9-CM: 401–405), hyperlipidemia (ICD-9-CM: 272.0–272.4), diabetes (ICD-9-CM: 250), chronic obstructive pulmonary disease (ICD-9-CM: 490–496), chronic kidney disease (ICD-9-CM: 585), and stroke (ICD-9-CM: 430–438). Those comorbidities were defined before the index date within one year.

**Figure 1:**
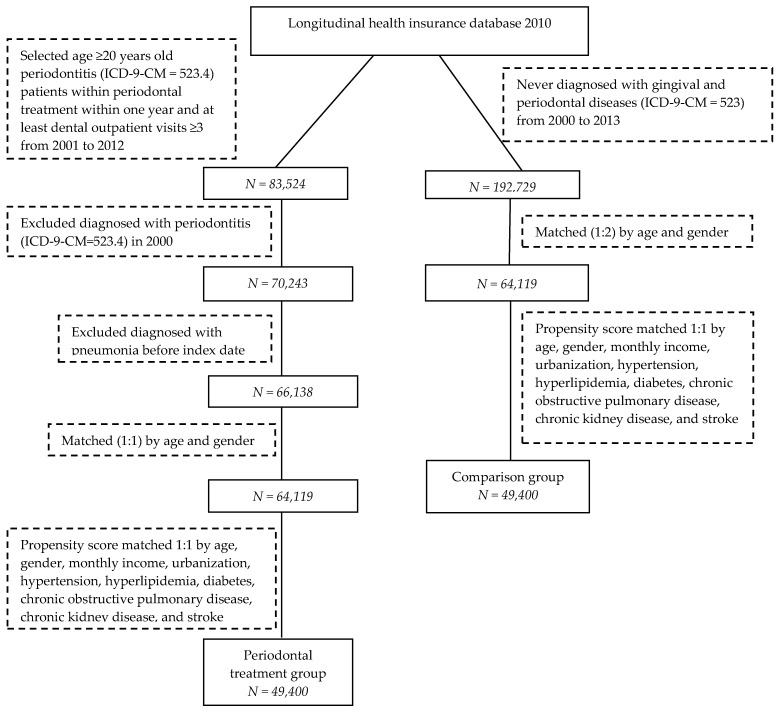
Flow diagram of patients’ enrollment in the study cohorts.

The comparison cohort was then frequency-matched according to age and gender at a 1:2 ratio in order to provide the same index date for both groups. During matching, we excluded patients who received a diagnosis of pneumonia before the index date. Moreover, propensity score matching (1:1) was performed for the comparison group according to age, gender, monthly income, urbanization, hypertension, hyperlipidemia, diabetes, chronic obstructive pulmonary disease, chronic kidney disease, and stroke. The propensity score was a probability that was calculated from logistic regression. By matching the propensity score, the difference in disease severity between the two groups could be reduced. The flow chart of case selection and exclusion is shown in Figure 1.

### 2.4. Pneumonia Event

The outcome was defined as newly diagnosed pneumonia (ICD-9-CM: 481, 482, 483, 485, and 486) from emergencies or hospitalization to confirm the accuracy of diagnosis. All patients were traced until the occurrence of pneumonia, withdrawal from the social insurance system, or the end of 2013, whichever came first.

### 2.5. Statistical Analysis

The Student’s t-test and Chi-square test were used to compare the demographic and clinical characteristics of patients with periodontal treatment with those of the comparison groups. The Kaplan–Meier analysis was used to plot the cumulative incidence of pneumonia. The log-rank test was used to compare differences between these two cohorts. The Cox proportional hazard models were applied to estimate the hazard ratios and 95% confidence intervals of periodontal treatment. All statistical analyses were performed using SPSS version 18 (SPSS, IBM, Chicago, IL, USA). The level of statistical significance was set at *p* < 0.05.

## 3. Results

The baseline demographic data of this study are listed in Table 1. In total, 49,400 patients with periodontal treatment and 49,400 people in the comparison group with similar age, income, and urbanization distributions were selected in this study (*p* > 0.05). There were no significant potential differences in comorbidities between the periodontal treatment group and the comparison group (*p* > 0.05).

A total of 1922 and 2504 patients were newly diagnosed with pneumonia in the periodontal treatment cohort and comparison cohort, respectively. The incidence density rate of pneumonia in the periodontal treatment group was only 5.1 per 1000 person-years (Table 2). The incidence density rate was about 0.75-fold lower in the periodontal treatment group than in the comparison group (6.8 per 1000 person-years). Patients with periodontal treatment had a lower risk of pneumonia as compared with the comparison group (adjusted HR: 0.69; 95%CI: 0.65–0.73). The age-specific adjusted HR of pneumonia increased with age from 2.28 (age 40–64 years) to 7.98 (age ≥ 65 years) as compared with the age 20–39 group. The male group had a higher pneumonia risk than the female group (adjusted HR: 1.60; 95%CI: 1.51–1.71). The higher monthly income group had a lower risk for pneumonia (adjusted HR: 0.65; 95%CI: 0.58–0.73). Those living in rural areas had a higher risk of pneumonia (adjusted HR: 1.18; 95%CI: 1.07–1.31). In addition, patients with hypertension, diabetes, chronic obstructive pulmonary disease, chronic kidney disease, and stroke demonstrated a significant risk of pneumonia (*p* < 0.05).

**Figure 2:**
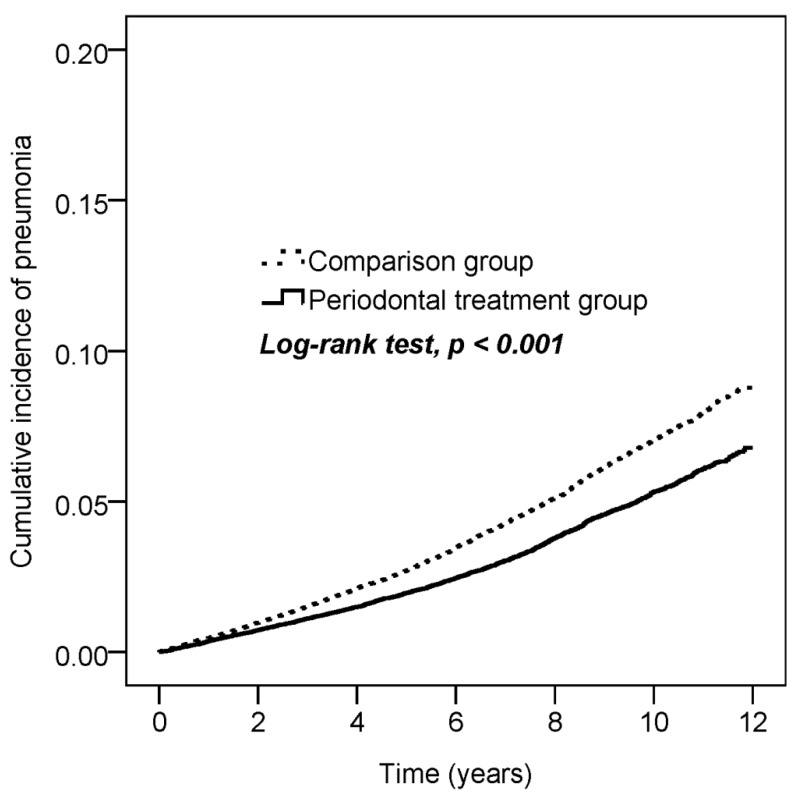
Kaplan–Meier plot for the cumulative incidence of pneumonia in the periodontal treatment group and comparison group used in this cohort study.

As shown in Figure 2, patients who received periodontal treatment had a significantly lower cumulative incidence of pneumonia than subjects in the comparison group over the 12 year follow-up period (using the log-rank test, *p* < 0.001).

As shown in Table 3, the mean follow-up duration in the periodontal treatment and comparison cohorts was 7.66 years and 7.41 years, respectively. The mean interquartile range for pneumonia was 5.34 and 5.16 years for the periodontal treatment cohort and the comparison cohort, respectively.

Further stratification with respect to the type of periodontal treatment, shown in Table 4, indicated that scaling could significantly reduce the risk of pneumonia (adjusted HR: 0.70; 95% CI: 0.66–0.75) compared to lack of periodontal treatment. In addition, intensive periodontal treatment such as flap surgery could lower pneumonia risk about 66% compared with lack of periodontal treatment (adjusted HR: 0.34; 95%CI: 0.19–0.62).

## 4. Discussion

Although several studies have reported the association of periodontal disease and pneumonia [17,18,19], little is known about the relationship between periodontal treatment and pneumonia risk. To the best of our knowledge, this is the first nationwide population-based cohort study reporting that the risk of pneumonia in patients who received periodontal treatment was lower than in the untreated group. Similar results were found by Yoneyama et al. who reported that intervention promoting oral hygiene could reduce pneumonia in elder inpatients and patients in nursing homes [20]. Recently, intensive periodontal treatment was associated with a 29% reduction in the risk of hospitalization for pneumonia in hemodialysis patients [21]. These data demonstrate that dental prophylaxis is very important for the reduction of pneumonia.

The oral cavity is an important source of bacteria that cause infections of the respiratory system. The connection of periodontal diseases and pneumonia might result from the colonization of pathogenic bacteria present in dental biofilm [22], followed by aspiration of the colonized pathogens, which is considered a significant risk factor for pneumonia [23,24]. Various respiratory pathogens such as *Staphylococcus aureus*, *Pseudomonas aeruginosa*, *Acinetobacter baumannii*, and *Enterobacter cloacae* were found in the dental biofilm of susceptible individuals [25,26,27]. In addition, many periodontal bacteria species have been cultured from infected lung fluids such as *Porphyromonas gingivalis*, *Eikenella corrodens*, *Fusobacterium nucleutum,* and *Actinomyces* [28,29]. These findings are a direct evidence of the positive association between periodontal disease and pneumonia. Therefore, dental biofilm could serve as an important reservoir of respiratory pathogens.

The monthly income and urbanization of the locations of NHI registration were used as a proxy parameter for socioeconomic status. It is not surprising that our results found that higher income and level of urbanization were associated with a relatively lower risk of pneumonia. This might be due to the larger availability of dental service and favorable patients’ attitudes toward periodontal treatment in those areas. These factors would influence people’s utilization of the NHI in Taiwan. However, further assessments such as through questionnaires and surveys are required. 

Poor oral hygiene in dentate patients was found to cause significantly higher salivary bacterial counts than in individuals with good oral hygiene [30]. Poor oral hygiene could increase the complexity and mass of the dental biofilm, enhancing further interactions between oral and respiratory pathogens [31]. In clinical dentistry, scaling, root planing, and flap surgery with oral hygiene instruction are the guidelines for regular periodontal treatment. These procedures can effectively remove dental biofilm and calculus from teeth surfaces and reduce inflammatory conditions, improving periodontal health. This may explain why periodontal treatment could reduce the risk of pneumonia in this study. Moreover, intensive periodontal treatment such as root planing and flap surgery were revealed to be more powerful in lowering the pneumonia risk than dental prophylaxis by scaling.

The strengths of this study are the use of a large, longitudinal sample size to evaluate the pneumonia risk in the periodontal treatment and comparison groups. The cohort study design used could confer a higher level of evidence to demonstrate a causal relationship between periodontal treatment and the decrease of the risk of pneumonia.

However, some potential limitations of this observational study should be addressed. First, this study was not a prospective, randomized study of chronic periodontitis patients who did or did not receive adequate periodontal treatment. Future study with controlled interventions is required. Second, we are uncertain about the periodontal health status of the comparison cohort due to this being a register-based study. Only individuals sought for dental prophylaxis and periodontal treatment could be recruited in the NHI system. Therefore, the exclusion of patients with any gingival and periodontal disease from 2000 to 2013 in the comparison group could reduce the selection bias and avoid the confounding variates. This is the reason why we chose individuals without periodontal diseases diagnoses (ICD-9-CM code 523) as the comparison cohort. Thirdly, the contributions of smoking habits, alcohol use, family history, or nutrition status factors could not be investigated using LHID2010. Fourthly, all of the diagnoses used ICD-9-CM codes in the NHIRD. Ventilator-associated pneumonia is a major infection in hospitals. The severity, pathogens, or causes of pneumonia were not reported in this database. Fifthly, the definitive code of aspiration pneumonia was included in ICD-9; however, aspiration pneumonia is a dynamic disease, and we could not distinguish between aspiration pneumonia and infectious pneumonia from the claims-based insurance database. These may compromise our findings and result in an inadequate adjustment of confounding factors. Finally, in this study, the majority of the participants were Taiwanese. It still remains uncertain whether the findings could be generalized to other ethnic groups.

## 5. Conclusions

In conclusion, this nationwide cohort study suggests that patient with periodontal treatment is associated with a decreased risk for pneumonia. Dentists and medical doctors should be more aware of the correlation of periodontal diseases and pneumonia. These findings could provide evidence to policy-makers that periodontal treatment is a potential modifiable factor in the primary prevention of pneumonia.

## Figures and Tables

**Table 1:** Demographic data of matched study cohorts.

	Periodontal Treatment Group (N = 49,400)		Comparison Group (N = 49,400)		
	*n*	%	*n*	%	*p*-Value
Age					<0.001
20–39	20,500	41.5	21,279	43.1	
40–64	23,838	48.3	23,295	47.2	
≥65	5062	10.2	4826	9.8	
Mean ± SD	44.25 ± 14.82		44.15 ± 14.5		0.283
Gender					0.049
Female	26,242	53.1	26,551	53.7	
Male	23,158	46.9	22,849	46.3	
Monthly income					0.007
<NT $20,000	24,560	49.7	25,021	50.6	
NT $20,000–NT $40,000	17,660	35.7	17,444	35.3	
>NT $40,000	7180	14.5	6935	14.0	
Urbanization					0.797
Urban	31,674	64.1	31,750	64.3	
Suburban	14,381	29.1	14,352	29.1	
Rural	3345	6.8	3298	6.7	
Hypertension	5761	11.7	5505	11.1	0.010
Hyperlipidemia	1997	4.0	1973	4.0	0.697
Diabetes	2651	5.4	2527	5.1	0.077
Chronic obstructive pulmonary disease	1367	2.8	1326	2.7	0.423
Chronic kidney disease	214	0.4	233	0.5	0.368
Stroke	887	1.8	872	1.8	0.718

The Student’s t-test and Chi-squared test were used to test the difference of continuous and categorical variables, respectively.

**Table 2:** Risk factor analysis of pneumonia development.

	No. of Event	Observed Person-Years	ID	Crude HR	95% CI	Adjusted HR ^†^	95% CI
Group							
Comparison	2,504	366,251	6.8	1		1	
Periodontal treatment	1922	378,522	5.1	0.74	0.70–0.78	0.69	0.65–0.73
Age							
20–39	703	325,108	2.2	1		1	
40–64	1889	351,970	5.4	2.51	2.30–2.73	2.28	2.09–2.50
≥65	1834	67,695	27.1	12.95	11.87–14.13	7.98	7.24–8.79
Gender							
Female	1800	400,036	4.5	1		1	
Male	2626	344,737	7.6	1.70	1.60–1.80	1.60	1.51–1.71
Monthly income							
<NT $20,000	2508	373,501	6.7	1		1	
NT $20,000–NT $40,000	1558	262,588	5.9	0.89	0.83–0.94	0.91	0.85–0.97
>NT $40,000	360	108,685	3.3	0.49	0.44–0.55	0.65	0.58–0.73
Urbanization							
Urban	2589	478,277	5.4	1		1	
Suburban	1390	216,786	6.4	1.18	1.11–1.26	1.05	0.98–1.12
Rural	447	49,710	9.0	1.66	1.50–1.84	1.18	1.07–1.31
Hypertension	1403	76,450	18.4	4.18	3.93–4.46	1.42	1.31–1.53
Hyperlipidemia	360	25,386	14.2	2.63	2.36–2.92	0.95	0.85–1.07
Diabetes	729	34,065	21.4	4.27	3.94–4.62	1.78	1.63–1.94
Chronic obstructive pulmonary disease	431	19,815	21.8	3.95	3.57–4.36	1.77	1.6–1.96
Chronic kidney disease	103	2577	40.0	7.25	5.96–8.82	2.69	2.21–3.28
Stroke	361	11,462	31.5	5.88	5.28–6.55	1.74	1.55–1.95

Bold font represents statistical significance (*p* < 0.05). ID: Incidence density, per 1000 person-years. † Adjusted for age, gender, monthly income, urbanization, hypertension, hyperlipidemia, diabetes, chronic obstructive pulmonary disease, chronic kidney disease, and stroke.

**Table 3:** Track time of the periodontal treatment and comparison cohorts.

	Periodontal Treatment Group (N = 49,400)	Comparison Group (N = 49,400)	*p*-Value
Follow-up duration (years)	7.66 ± 3.05	7.41 ± 3.14	<0.001
Time to event (years), N = 4426	5.34 ± 3.07	5.16 ± 3.01	0.057

The Student’s t-test was used to test the difference between continuous variables.

**Table 4:** Hazard ratios of pneumonia development among different periodontal treatments.

	N	No. of Event	Crude HR	95% CI	Adjusted HR ^†^	95% CI
Periodontal treatment						
None	49,400	2504	1		1	
Scaling	44,253	1783	0.74	0.7–0.79	0.70	0.66–0.75
Root planing	4380	128	0.76	0.64–0.91	0.58	0.48–0.69
Flap surgery	767	11	0.35	0.19–0.63	0.34	0.19–0.62

† Adjusted for age, gender, monthly income, urbanization, hypertension, hyperlipidemia, diabetes, chronic obstructive pulmonary disease, chronic kidney disease, and stroke.
